# MK591, a Second Generation Leukotriene Biosynthesis Inhibitor, Prevents Invasion and Induces Apoptosis in the Bone-Invading C4-2B Human Prostate Cancer Cells: Implications for the Treatment of Castration-Resistant, Bone-Metastatic Prostate Cancer

**DOI:** 10.1371/journal.pone.0122805

**Published:** 2015-04-15

**Authors:** Sivalokanathan Sarveswaran, Ritisha Ghosh, Shravan Morisetty, Jagadananda Ghosh

**Affiliations:** 1 Department of Urology, Henry Ford Health System, Detroit, MI 48202, United States of America; 2 Josephine Ford Cancer Center, Henry Ford Health System, Detroit, MI 48202, United States of America; UC Davis Comprehensive Cancer Center, UNITED STATES

## Abstract

Castration-resistant prostate cancer (CRPC) is a major clinical challenge for which no cure is currently available primarily because of the lack of proper understanding about appropriate molecular target(s). Previously we observed that inhibition of 5-lipoxygenase (5-Lox) activity induces apoptosis in some types of prostate cancer cells, suggesting an important role of 5-Lox in the viability of prostate cancer cells. However, nothing is known about the role of 5-Lox in the survival of castration-resistant, metastatic prostate cancer cells. Thus, we tested the effects of MK591, a second-generation, specific inhibitor of 5-Lox activity, on the viability and metastatic characteristics of CRPC cells. We observed that MK591 effectively kills the bone-invading C4-2B human prostate cancer cells (which bear characteristics of CRPC), but does not affect normal, non-cancer fibroblasts (which do not express 5-Lox) in the same experimental conditions. We also observed that MK591 dramatically inhibits the *in vitro* invasion and soft-agar colony formation of C4-2B cells. Interestingly, we found that treatment with MK591 dramatically down-regulates the expression of c-Myc and its targets at sub-lethal doses. In light of frequent over-activation of c-Myc in a spectrum of aggressive cancers (including CRPC), and the challenges associated with inhibition of c-Myc (because of its non-enzymatic nature), our novel findings of selective killing, and blockade of invasive and soft-agar colony-forming abilities of the castration-resistant, bone-metastatic C4-2B prostate cancer cells by MK591, open up a new avenue to attack CRPC cells for better management of advanced prostate cancer while sparing normal, non-cancer body cells.

## Introduction

The American cancer society estimates that during the year 2014, about 233,000 new cases of prostate cancer will be diagnosed in the United States and about 29,480 will die from the disease which makes prostate cancer the most common form of malignancy and second-leading cause of cancer-related deaths in American men [1]. Considerable advances in screening and diagnosis allowed detecting prostate cancer at early stage for which the therapeutic options are curative including surgery and radiation [2–4]. Current androgen deprivation therapy by decreasing circulating testosterone effectively shrinks androgen-dependent prostate tumors. However, in spite of initial positive response, most men ultimately fail to this therapy. Moreover, continuous androgen deprivation usually leads to recurrent castration-resistant prostate cancer which is a major clinical challenge in the management of prostate cancer [5,6]. CRPC patients who have failed hormone deprivation therapy are treated with standard docetaxel-based combination chemotherapy. However, only limited improvement in survival was observed in these patients at the cost of a huge compromise with the quality of life [7], and there is no effective therapy for disseminated, late-stage prostate cancer. Since, the development of CRPC is causally linked to high prostate cancer mortality, now a great deal of emphasis is on development of new therapeutic strategies for the management of CRPC. However, lack of proper understanding about the biology of CRPC cells is hampering the development of effective therapies which are urgently needed in the clinic.

A key to the development of effective therapy against CRPC is the identification and characterization of molecular targets which play critical roles in the survival and growth characteristics of CRPC cells. Epidemiological studies and animal experiments repeatedly suggested a link between high-fat diets and occurrence of clinical prostate cancer [8–13]. Moreover, a strong association between arachidonic acid and the risk of metastatic prostate cancer has been reported [14–16]. We and others have observed that, arachidonic acid, an omega-6 polyunsaturated fatty acid, promotes growth and survival of prostate cancer cells via metabolic conversion through the 5-Lox pathway [17–19]. Previous studies in our laboratory documented that prostate cancer cells continuously generate 5-Lox metabolites, and inhibition of 5-Lox blocks production of 5-Lox metabolites and induces apoptosis [20,21]. Interestingly, inhibition of 5-Lox was found to kill both androgen-receptor positive as well as androgen-receptor negative prostate cancer cells. Also, prevention of apoptosis by exogenous 5-Lox metabolite 5(S)-HETE and more effectively by its dehydrogenase-derivative 5-oxoETE strongly suggest that 5-Lox plays a critical role in the survival of prostate cancer cells. Recently, we have found that 5-Lox inhibition-induced apoptosis occurs via down-regulation of PKCε, without inhibiting AKT or ERK (which are also characterized as regulators of pro-survival mechanisms), suggesting the existence of an AKT-and ERK-independent survival mechanism in prostate cancer cells regulated by 5-Lox activity [22,23].

Based on previous studies in our laboratory which demonstrated that inhibition of 5-Lox kills a range of prostate cancer cell lines without regard to their androgen receptor status, we hypothesized that 5-Lox may be a potential target for therapy of heterogeneous lethal forms of prostate cancer as well. Hence, we wanted to further analyze the effect of 5-Lox inhibition on the LNCaP human prostate cancer cell-derived C4-2B cells which still retain the androgen receptor, but are refractory to androgen stimulation. These cells were isolated from metastatic prostate cancer lesions found in the lumbar spine of athymic murine host and closely mimic cellular features of clinical bone-metastatic prostate cancer, and when injected orthotopically, C4-2B cells produce osteoblastic metastases in the lumbar bone [24]. Thus, the C4-2B cell line provides us with a model to explore potential effects of 5-Lox inhibitors for therapy of CRPC. In the present study, we observed that MK591, a second-generation, specific inhibitor of 5-Lox activity [25–29], effectively blocks the *in vitro* invasion and soft-agar colony formation, and triggers apoptosis in C4-2B prostate cancer cells in a dose and time-dependent manner. Moreover, we observed that MK591 activates phosphorylation of c-JNK, induces mitochondrial permeability transition, and triggers degradation of chromatin DNA to nucleosomal fragments. Interestingly, while MK591 exerts a strong effect on C4-2B prostate cancer cells, it does not kill normal, non-cancer human foreskin fibroblasts (HFF) which do not express 5-Lox in them. Altogether, these findings suggest that the 5-Lox inhibitor, MK591, may be effective for prevention and therapy of castration-resistant, bone-metastatic, lethal form of prostate cancer.

## Materials and Methods

### 2.1. Cell culture and reagents

Normal human foreskin fibroblasts and C4-2B human prostate cancer cells were purchased from American Type Culture Collection (Manassas, VA). Fibroblasts and C4-2B cells were grown in DMEM and RPMI-1640 (Invitrogen, Carlsbad, CA) with 10% fetal bovine serum respectively. All the media were supplemented with 100 U/ml penicillin and 100 μg/ml streptomycin and cell cultures were maintained in a humidified atmosphere. Antibodies against PARP, PKCε, cyclin D1, CDK4, Bcl-2 and XIAP were from Santa Cruz Biotechnology (Santa Cruz, CA). Polyclonal anti-5-Lox antibody was purchased from ProteinTech (Chicago, IL). Anti-survivin antibody was purchased from R&D systems (Minneapolis, MN). Polyclonal antibodies against Akt, phospho-Akt (Ser^473^), phospho-JNK (Thr^183/Y185^) and JNK were purchased from cell Signaling Technology (Danvers, MA). Anti-beta-actin antibody, docetaxel and ibuprofen were purchased from Sigma Chemical CO (St. Louis, MO). SP600125 and U0126 were purchased from Calbiochem (San Diego, CA). MK591 was obtained as a generous gift from Dr. Robert N. Young (Merck-Frosst Centre for Therapeutic Research, Quebec, Canada).

### 2.2 Microscopy

C4-2B cells (~3 x 10^5^) were plated in RPMI-1640 medium supplemented with 10% FBS onto 60 mm diameter tissue culture plates (Falcon) and allowed to grow for 48 h. On the day of experiment, the spent culture medium was replaced with 2 ml fresh RPMI medium with 10% FBS and cells were treated with inhibitors. Control cells were treated with the solvent (DMSO) only. HFF were plated in DMEM and treated with inhibitors in the same way as C4-2B cells. After 72 hours, photographs were taken with a Nikon digital camera attached to a LEICA fluorescence microscope at x200. Image acquisition and data processing were done with a DELL computer attached to the microscope using Q-Capture Pro7 software.

### 2.3. Cell viability assay

C4-2B prostate cancer and human foreskin fibroblast cells (~3000 per well) were plated overnight in 96 well plates in complete growth medium (RPMI or DMEM plus 10% FBS) and treated with varying doses of MK591. Plates were incubated further for 72 h at 37 °C in the CO_2_ incubator. Cell viability was measured by One Solution MTS/PES Cell Titer assay from Promega (Madison, WI) as described before [17,23].

### 2.4. Annexin-V binding

C4-2B prostate cancer cells (~3×10^5^) were plated in RPMI medium and allowed to grow for 48 h. The spent culture medium was replaced with fresh 2 ml RPMI medium and the cells were treated with MK591 or ibuprofen for 24 h at 37°C. Then the cells in the plate were treated with FITC-labeled annexin-V and propidium-iodide (PI) for 15min in the dark following a protocol supplied by the manufacturer with the Annexin V-Binding Detection Kit (BD Biosciences). After washing, cells were photographed with a Nikon digital camera attached to a Leica fluorescence microscope at x200. Image acquisition and data processing were done with a Dell computer attached to the microscope using Q-Capture Pro7 software.

### 2.5. Western blot

C4-2B cells (~3×10^5^) were plated in 60 mm diameter plates and allowed to grow for 48 hours. The old medium was then replaced with 2 ml fresh RPMI medium and the cells were treated with inhibitors. After treatment, cells were harvested, washed, and lysed in lysis buffer (50 mM HEPES, pH 7.4, 150 mM NaCl, 1 mM EDTA, 1 mM orthovanadate, 10 mM sodium pyrophosphate, 10 mM sodium-fluoride, 1% NP-40, and a cocktail of protease inhibitors). Proteins were separated by 12% SDS-PAGE and transferred to nitrocellulose membranes. Membranes were blocked with 5% non-fat milk solution and then blotted with appropriate primary antibody followed by horseradish peroxidase-labeled secondary antibody. Bands were visualized by enhanced chemiluminescence detection kit from Pierce Biotech (Rockford, IL). Unless otherwise mentioned, blots of proteins of interest were analyzed in three separate experiments.

### 2.6. Measurement of DNA degradation

Apoptosis was quantitatively measured by detecting degradation of nuclear DNA to nucleosomal fragments by sandwich-ELISA. C4-2B cells (~3×10^5^) were plated in 60 mm dishes and allowed to grow for 48 h. Then, the cells were treated either with the experimental agents at varying concentrations or the solvent vehicle for 24 h. At the end of incubation periods, cells were harvested, lysed and the degradation of chromatin-DNA to nucleosomal fragments was measured by a Cell Death Detection ELISA ^plus^ kit from Roche (Indianapolis, IN) as described before [20–23].

### 2.7. Measurement of mitochondrial trans-membrane potential

C4-2B cells (~3 x 10^5^) were plated in 60 mm diameter culture dishes (Falcon) for 48 hours and then treated either with experimental agent or solvent vehicle for 16 hours at 37°C. Then the cells in the plate were treated with Mito-Tracker Red Dye for 15 minutes in the dark using Vybrant Apoptosis assay kit following a protocol supplied by the manufacturer (Molecular Probes). Then, the cells were counterstained with blue-fluorescent DAPI DNA-binding dye (Sigma). After washing, cells were photographed with a Nikon digital camera attached to a Leica fluorescence microscope at x200. Image acquisition and data processing were done with a Dell computer attached to the microscope using Q-Capture Pro7 software.

### 2.8. Boyden chamber invasion assay

Matrigel inserts (BD Biosciences, Sparks, MD) were adapted for room temperature and rehydrated with 50 μl serum-free RPMI-1640 medium. Then, ~50,000 C4-2B cells (re-suspended in 100 μl serum-free RPMI with different concentration of inhibitors) were carefully seeded on the soaked matrigel in the upper chamber. The lower chamber was filled with 500 μl RPMI plus 2% serum and inhibitors. Then the matrigel invasion chambers were incubated for 16 hours at 37°C in the CO_2_ incubator. After incubation, non-invading cells are removed from the upper surface of the membrane by scrubbing with sterile cotton swab moistened with medium. Then the cells were fixed by 70% ethanol and stained with crystal violet. After air dry, membrane from the inserts were removed and mounted on microscope slides. Stained cells were then photographed at x400, and the total number of invaded cells counted in triplicates.

### 2.9. Reverse-transcriptase polymerase chain reaction (RT-PCR)

Total RNA was isolated from confluent cultures of HFF (human foreskin fibroblasts) and C4-2B cells using RNeasy Mini kit (Qiagen, Maryland, USA). cDNA was synthesized using 2 μg of total RNA with SuperScript III First-Stand Synthesis Supermix (Invitrogen). The primers for 5-Lox were 5’-CCCGGGGCATGGAGAGCA-3’ (Forward) and 5’-GCGGTCGGGCAGCGTGTC-3’ (Reverse). The beta actin primers were 5’-CTCCTGCTTGCTGATCCACAT-3’ (forward) and 5’-AACCGCGAGAAGATGACCCAG-3’ (Reverse). The PCR conditions were as follows, For 5-Lox: 1 min at 94°C, 36 cycles of 15 Sec at 94°C, 15 Sec at 60°C, 60 Sec at 72°C and 7 min at 72°C. For beta-actin: 1 min at 94°C; 30 cycles of 30 Sec at 94°C; 30 Sec at 59°C; 60 Sec at 72°C and 7 min at 72°C. The resulting PCR products were resolved and visualized by 1.5% agarose gel stained with ethidium bromide. Bands were photographed and analyzed with Eagle Eye II Dark-room Cabinet still video imaging system using Eagle-Sight v3.1 software (Stratagene, La Jolla, CA).

### 2.10. Real-time quantitative polymerase chain reaction (qPCR)

C4-2B cells were plated and treated with 30 μM MK591 at 37^°^C. Then, the cells were harvested, washed and RNA was isolated from exponentially growing cells using Qiagen RN-Easy Mini Kit from Qiagen. For the real-time PCR, one microgram of total RNA was used for the RT reaction using high capacity cDNA-RT kit from ABI/Life Technologies. Then the qPCR reactions were performed using TaqMan gene expression assay kits from ABI/Life Technologies using ABI-7500 Fast real-time qPCR machine.

### 2.11. Soft-agar colony formation assay

Colony formation assays were performed in six well tissue culture plates by placing ~5,000 C4-2B cells in 0.5 ml of 0.3% soft-agar on top of a 2 ml base layer of 0.6% agar. Plates were allowed to settle and then the wells were covered with 2 ml fresh RPMI medium containing 10% FBS with or without inhibitors. Then, the plates were incubated at 37^°^C in the CO_2_ incubator for a period of three weeks. Cell growth medium and inhibitors were exchanged every fourth day. At the end of incubation period, cells were stained with 0.25% crystal violet in PBS for 30 min and photographed with a Nikon digital camera attached to a Leica microscope at x300. Then, the total number of colonies (with ≥ 50 cells per colony) in each well was counted for quantitation.

### 2.12. Luciferase assays

C4-2B cells were transfected with lentiviral Stat3-luciferase or E-box-luciferase constructs (>90% cells transfected), expanded, and re-plated in 96 well culture plates in triplicates. Cells were then treated with inhibitors (MK591 or Stattic) with proper controls and the luciferase activities were measured by a luciferase assay kit (Bright-Glo) from Promega Corporation (Madison, WI).

## Results

### 3.1. MK591 selectively decreases viability of the castration-refractory, bone-invading C4-2B prostate cancer cells

**Fig 1:**
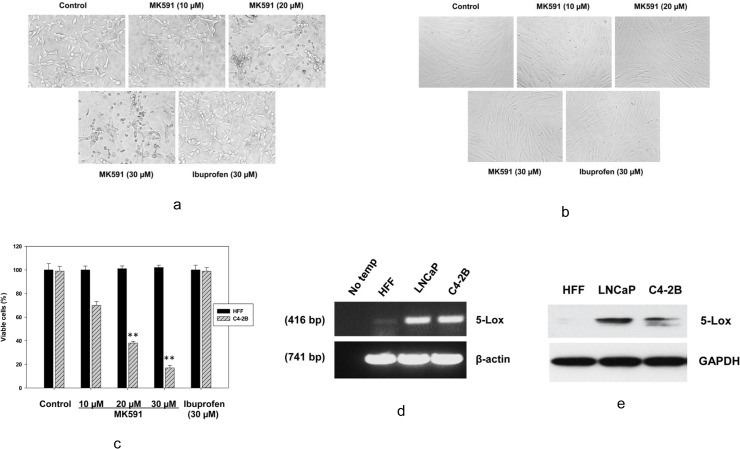
Effects of MK591 on the morphology and viability of C4-2B cells. C4-2B prostate cancer cells and normal HFF cells (~3 x 10^5^ cells per plate) were plated in 60 mm diameter plates and allowed to grow for 48 hours. Then the cells were treated with varying doses of MK591 for 72 hours at 37^°^C in the incubator. Ibuprofen (a cyclooxygenase inhibitor) was used as negative control. At the end of incubation period, photographs were taken with a Nikon digital camera attached to a Leica microscope at x400 and processed on a Dell computer using Q-Capture Pro7 software. In (c), comparative effects of MK591 on the viability of cancer (C4-2B) and normal (HFF) cells are shown after treating cells under the same experimental conditions. Control cells were treated with vehicle only (0.2% DMSO). At the end of treatment period, cell viability was measured by MTS/PES assay as described before (20–23). Results are quantitatively presented as mean values of each data point ± standard error (n = 4). ** p = <0.005. In (d and e), Expression of 5-Lox in HFF and C4-2B cells was detected by RT-PCR and Western blot as described in the “Methods” section.

Previously we found that both MK591 and 5-Lox shRNAs decrease the viability of LNCaP prostate cancer cells via induction of apoptosis [23]. This apoptosis is prevented by metabolites of 5-Lox, suggesting that 5-Lox activity plays an important role in the survival of these cancer cells. Later, we found that the active 5-Lox metabolite (5-oxoETE) signals via its cognate GPCR, OXER1, and involves PKC-epsilon as down-stream kinase [30]. Since most of the deaths due to prostate cancer happen due to androgen-resistant, metastatic disease, we wanted to examine whether MK591 can affect the bone-invading C4-2B prostate cancer cells which are androgen receptor positive but do not depend on androgenic-stimulus for their growth and survival. We observed that inhibition of 5-Lox by MK591 dramatically alters the morphology of C4-2B prostate cancer cells, whereas ibuprofen, an inhibitor of cyclooxygenase was ineffective, suggesting a role of 5-Lox in the survival of C4-2B cells (Fig 1A). Interestingly, we observed that MK591 did not decrease the viability of normal human foreskin fibroblasts (HFF) isolated from healthy donors (Fig 1B and 1C). Analysis of HFF and C4-2B cells by RT-PCR and Western blot revealed that whereas the C4-2B cells express high amounts of 5-Lox mRNA and protein, the expression of 5-Lox in HFF cells is undetectable (Fig 1D and 1E), suggesting that the expression and function of 5-Lox is cancer-specific.

### 3.2. MK591 blocks the *in vitro* invasion and soft-agar colony-forming abilities of C4-2B prostate cancer cells at sub-lethal doses

**Fig 2:**
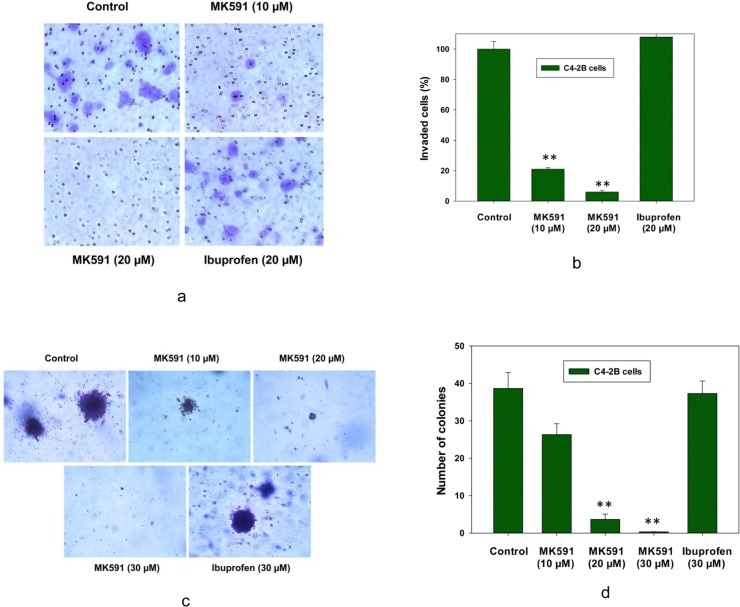
Effects of MK591 on in vitro invasion and soft-agar colony formation by C4-2B prostate cancer cells. In (a), the effect of MK591 on invasive capacity of C4-2B cells was assayed using matrigel-coated trans-well chambers as described in the “Methods” section. After incubation for 16 hours, cells were fixed and stained with crystal violet and pictures were taken with a Leica microscope at x300. In (b), quantitative measurements of the number of invaded cells are shown with or without drug treatment. Results represent mean values of individual data point ± standard deviation (n = 3), ** p = <0.005. In (c), effects of MK591 on soft-agar colony formation by C4-2B cells are shown. Cells (~5,000 per well in 6-well plate) were plated on soft-agar in RPMI medium as described in the “Methods” section. After incubation for three weeks, colonies were stained with crystal-violet and photographed under a Leica microscope at x300. In (d), growing colonies were counted under a microscope and the results are shown quantitatively as mean values of each data point ± standard deviation (n = 3). ** p = <0.005.

Castration-resistant, bone-metastatic prostate cancer cells are characterized by disproportionately higher invasive capability due to extensive genetic reorganization. This type of cancer is invariably lethal because no effective treatment is currently available to tackle their aggressive characteristics. Hence we tested the effect of MK591 on invasiveness of the androgen-refractory, bone metastatic C4-2B cells. This experiment was done using trans-well matrigel invasion chambers and cell culture inserts containing 8 micrometer pore-sized polyethylene terephthalate membrane coated with a thin layer of matrigel basement membrane matrix. We found that MK591 at sub-lethal doses dramatically block the *in vitro* matrigel-invasive capability and soft-agar colony-forming abilities of C4-2B prostate cancer cells, suggesting that MK591 may turn out to be an effective agent to control development and growth of castration-refractory prostate cancer with bone invasion (Fig 2A–2D). However, ibuprofen (a cyclooxygenase inhibitor) did not affect the invasive- or soft-agar colony-forming capabilities of C4-2B cells in the same experimental conditions at similar concentrations.

### 3.3. MK591 dramatically decreases the protein levels of c-Myc and its targets in C4-2B cells

**Fig 3:**
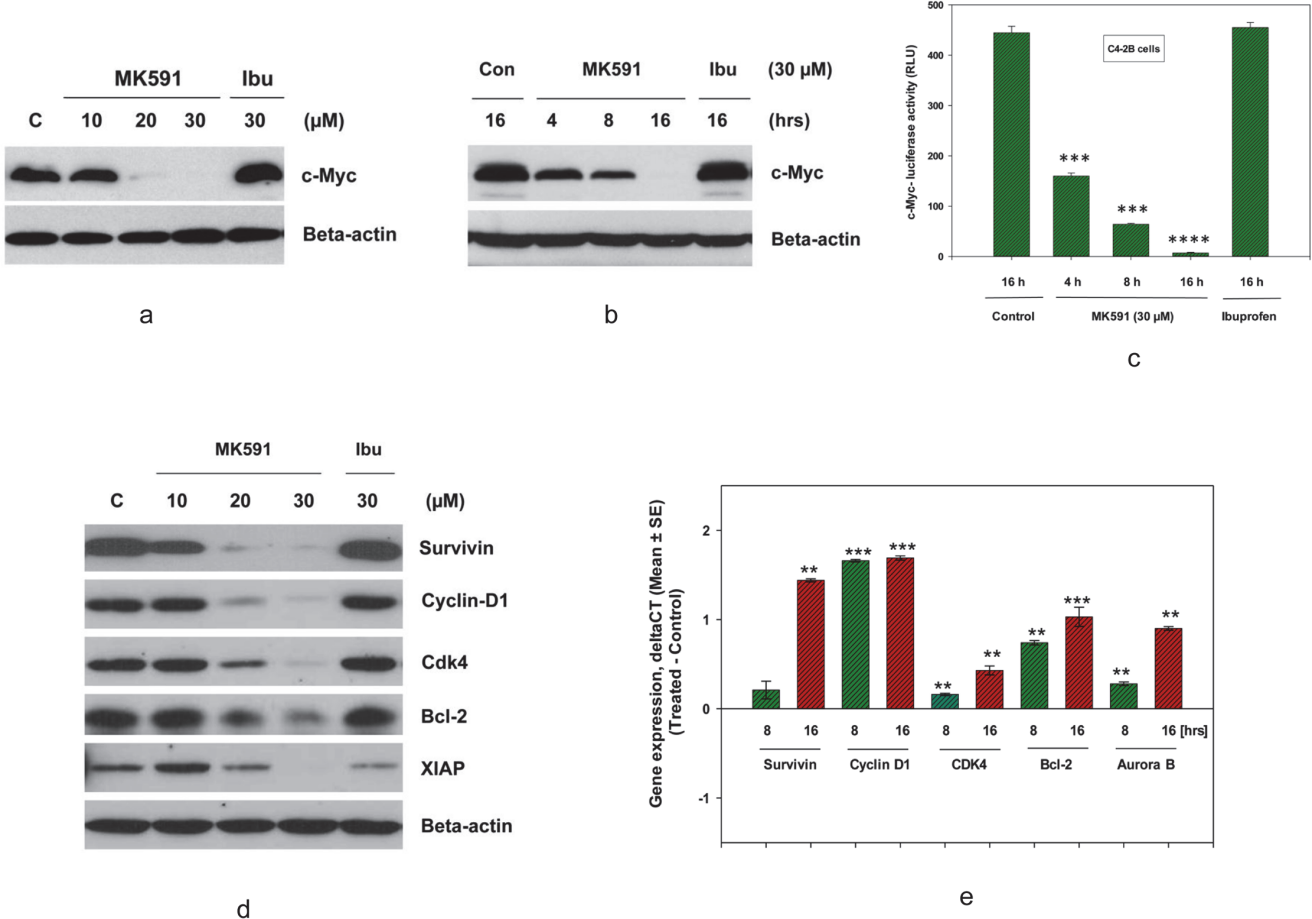
Effects of MK591 on protein levels of c-Myc and targets in C4-2B cells. C4-2B cells (~3 x 10^5^ cells per plate) were plated in 60 mm diameter plates and allowed to grow for 48 hours. Then, the old medium was replaced with 2 ml fresh RPMI medium and the cells were treated with MK591 and incubated for 24 hours at 37^°^C. In (a and b), dose- and time-dependent changes in the levels of c-Myc proteins by treatment with MK591 or ibuprofen are shown by Western blot. Beta-actin was used as loading control. In (c), effect of MK591 on c-Myc-luciferase activity was measured in a time-dependent manner. *** p = <0.0005 and **** p = <0.00005. In (d), effects of MK591 on protein levels of c-Myc-targets are shown by Western blot. ***Note*:** Ibuprofen, an inhibitor of cyclooxygenase (30 μM), was used in parallel which showed no appreciable effects on protein levels of either c-Myc or its targets. In (e), time-dependent decrease in mRNA expressions of c-Myc targets was detected by real-time PCR. Change in expression (delta-CT) was calculated by deducting mean values of control for each gene using the same probe set. Normalization of expression was done by using GAPDH as internal control.

The c-Myc oncoprotein plays a pivotal role in promoting metastasis in various types of cancer cells, including prostate cancer cells [31–40]. Thus, we analyzed the effect of MK591 treatment on the levels and functions of c-Myc as well as a range of its targets that are associated with prostate cancer cell survival, proliferation, and apoptosis-resistance. We found that MK591 decreases the protein level of c-Myc in a clear dose- and time-dependent manner (Fig 3A and 3B). Moreover, by using the c-Myc DNA-binding domain-luciferase constructs (E-box-Luc), we found that MK591 treatment reduced the transcriptional activity of c-Myc (Fig 3C). We also observed that both the protein levels as well as mRNA expression of a range of c-Myc transcriptional-targets are decreased under the same experimental conditions, suggesting that the transcriptional function of c-Myc in C4-2B cells is significantly inhibited by MK591 treatment (Fig 3D and 3E). Ibuprofen, a cyclooxygenase inhibitor, was used as a negative control which was found to be ineffective.

### 3.4. MK591 treatment-induced decrease in c-Myc levels in C4-2B cells occur via inhibition of Stat3-mediated transcription

**Fig 4:**
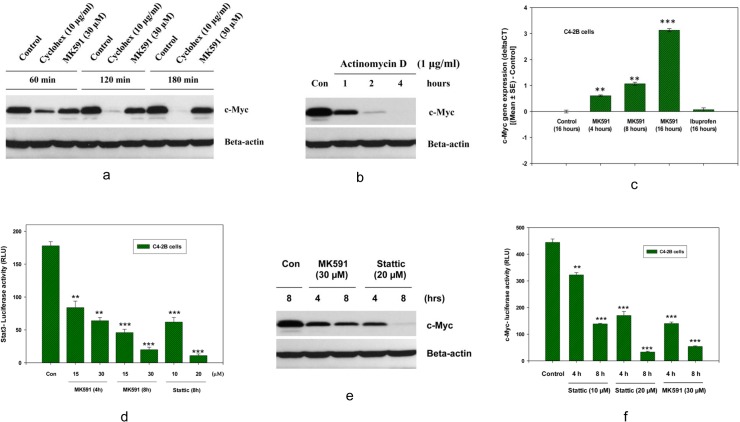
Involvement of the inhibition of Stat3-mediated transcription in MK591 treatment-induced decrease in c-Myc levels in C4-2B cells. C4-2B cells (~3 x 10^5^ cells per plate) were plated in 60 mm diameter plates and allowed to grow for 48 hours. Then, the old medium was replaced with 2 ml fresh RPMI medium and the cells were treated with cycloheximide or MK591 as indicated at 37^°^C. In (a), time-dependent changes in the levels of c-Myc proteins are shown by Western blot. Beta-actin was used as loading control. ***Note*:** MK591 treatment-induced decrease in c-Myc level is slower than cycloheximide treatment. In (b), time-dependent change in c-Myc protein level is shown after treatment of cells with Actinomycin D (a transcription inhibitor) only. In (c), effect of MK591 on c-Myc mRNA expression is shown by real-time PCR in a time-dependent manner. Note: A decrease in mRNA expression (~increased CT value) of c-Myc after MK591 treatment was detected by calculating the change in expression (delta-CT) deducting control values using the same PCR probe set. Normalization of expression was done by using GAPDH as internal control. In (d), effect of MK591 on Stat3-luciferase activity was measured in a time-dependent manner. Stattic (a Stat3 inhibitor) was used as positive control. ** p = <0.005 and *** p = <0.0005. In (e and f), effects of Stattic on protein level and transcriptional activities of c-Myc were detected by Western blot and E-box-luciferase assays respectively. ***Note*:** MK591 was also used in parallel experiments for side-by-side comparison with Stattic.

The dramatic loss of c-Myc protein due to MK591 treatment intrigued us to explore the underlying mechanism. At first, we performed a cycloheximide-chase experiment and found that the loss of c-Myc function by MK591 treatment is not due to enhanced protein degradation (Fig 4A). This finding triggered us to test the hypothesis whether the decrease in c-Myc protein after MK591-treatment happens due to a transcriptional blockade of c-Myc mRNA. We addressed this by treating cells with actinomycin D (a blocker of transcription), and found that actinomycin D dramatically decreases c-Myc protein level in similar time-frame, suggesting that a transcriptional blockade may reduce c-Myc protein level in these cancer cells (Fig 4B). Later, by real-time PCR, we confirmed that the expression of c-Myc mRNA decreases significantly with MK591 treatment (Fig 4C). We recently reported that the 5-lipoxygenase activity is linked with PKCε and Stat3 signaling in LNCaP cells [23]. Thus, we wanted to test whether MK591 reduces the level of c-Myc via Stat3-mediated transcription. Interestingly, we found that MK591 strongly inhibited the transcriptional activity of Stat3 in C4-2B cells (Fig 4D). Moreover, we found that Stattic (a Stat3 inhibitor) decreases both c-Myc protein level and c-Myc-luciferase activity in a clear dose- as well as time-dependent manner (Fig 4E and 4F), suggesting that MK591 treatment triggers disruption of c-Myc function in C4-2B cells via inhibition of Stat3-mediated transcription of the c-Myc gene.

### 3.5. MK591 induces membrane lipid-asymmetry, PARP-cleavage, caspase-3 activation, and DNA-degradation in C4-2B prostate cancer cells

**Fig 5:**
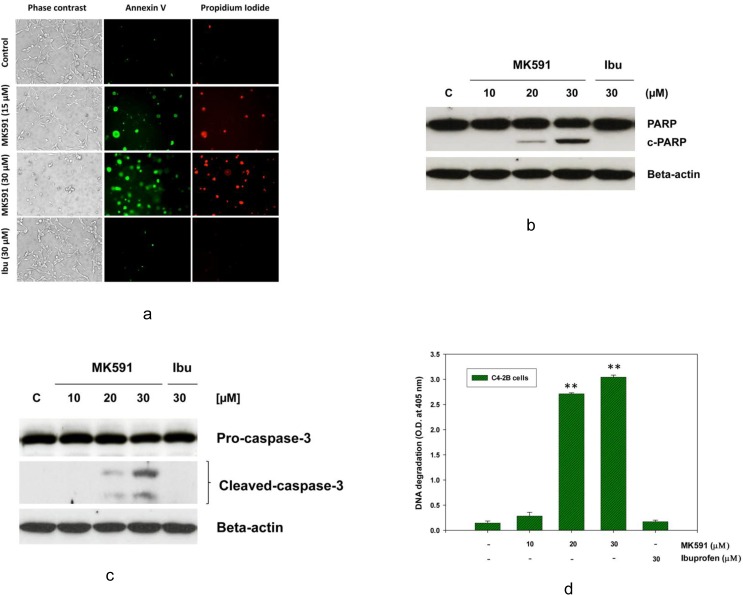
Induction of apoptosis in C4-2B cells by MK591 treatment. C4-2B cells (~3 x 10^5^ per plate) were plated in 60 mm diameter plates and allowed to grow for 48 hours. Then the old medium was replaced with 2 ml fresh RPMI medium and the cells were treated either with MK591 or ibuprofen for 24 hours at 37^°^C. Control cells were treated with vehicle only (0.2% DMSO). In (a), at the end of incubation period, intact cells were treated with FITC-labeled Annexin-V and propidium iodide (PI) in binding buffer and observed under microscope. Photographs were taken with a Nikon digital camera attached to a Leica fluorescence microscope at x200 and processed on a Dell computer using Q-Capture Pro7 software. In (b and c), at the end of incubation period, cells were lysed, and cleavage of PARP (c-PARP) and caspase-3 were detected by Western blot. In (d), degradation of nuclear DNA was detected by Cell Death Detection ELISA. Results are shown as mean values of each data point ± standard error (**p < 0.005, n = 4).

The morphological changes in C4-2B cells with MK591 treatment reminded us about the possibility of cells undergoing apoptosis. Indeed, we observed that treatment with MK591 induces externalization of phosphatidyl-serine (a hall-mark of apoptotic cell death) to the surface membrane which is characterized by distinctly positive binding of non-permeabilized C4-2B cells with annexin-V (Fig 5A). Characteristic cleavage of caspase-3 and PARP (poly-ADP ribose polymerase) which are indicators of caspase-mediated apoptosis were also observed in MK591 treated C4-2B cells in a dose-dependent manner (Fig 5B and 5C). Further analysis revealed elaborate degradation of nuclear DNA to nucleosomal fragments which is an indicator of advanced stage of apoptosis (Fig 5D). Interestingly, cells treated with ibuprofen in parallel experiments, did not show any signs of apoptotic features, suggesting that the induction of apoptosis in the C4-2B cells by the 5-Lox inhibitor is highly selective.

### 3.6. MK591 induces apoptosis in C4-2B cells via rapid activation of c-JNK

**Fig 6:**
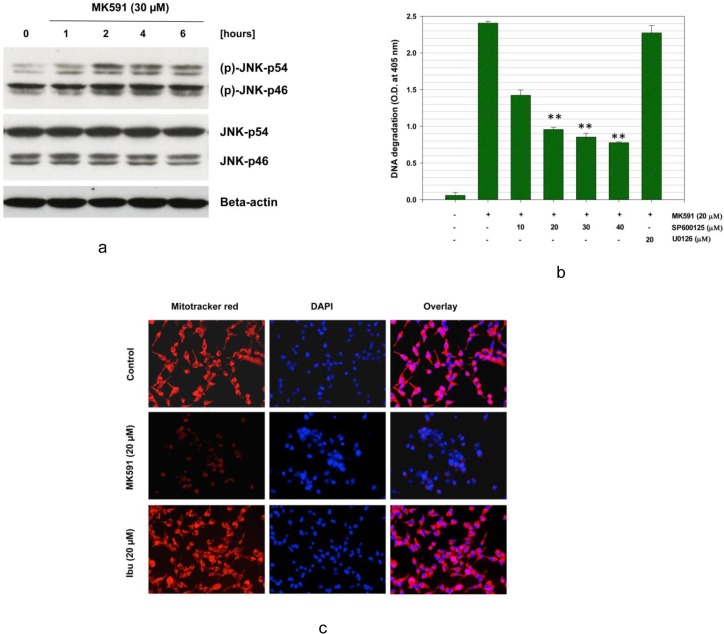
Activation and role of c-JNK in MK591-induced apoptosis. C4-2B cells were plated as in Fig 5 and treated with MK591 (30 μM) for varying periods of time and changes in the phosphorylation of c-JNK was detected by Western blot (a). Then, the membrane was stripped and re-probed with regular c-JNK antibody to detect total c-JNK protein. Beta-actin was used as loading control. In (b), to test the role of c-JNK in apoptosis, the cells were pre-treated with SP600125 (a c-JNK inhibitor) or U0126 (a MEKK inhibitor) for 30 minutes. Then, the cells were treated with MK591 and apoptosis was measured by sandwich ELISA. Results are shown as mean values of each data point ± standard error (**p < 0.005, n = 4). In (c), effect of MK591 on mitochondrial integrity was examined by treating cells with MK591 for 16 hours followed by mitotracker-red for 15 minutes in the dark. DAPI (blue) was used to stain the nuclei. Representative pictures are shown here from three independent experiments with similar results. ***Note*:** A dramatic loss of mitochondrial membrane-potential was observed with MK591 treatment while ibuprofen was found ineffective.

The c-Jun N-terminal Kinase (c-JNK) plays an important role in apoptosis induction in many cell types [41–47]. Hence, we wanted to know whether MK591 treatment activates c-JNK, and whether c-JNK plays any role in induction of apoptosis in these androgen-refractory, bone-metastatic C4-2B prostate cancer cells. We observed that a rapid activation of JNK occurs with MK591 which is distinguishable as early as one-hour post treatment (Fig 6A). Activation of c-JNK was detected by using dually-phosphorylated c-JNK antibodies (Thr 183-X-Tyr 185). Moreover, when the cells were pretreated with SP600125, a specific inhibitors of JNK activity, a dose dependent decrease in DNA degradation was observed, confirming that JNK plays an important role in MK591 treatment-induced apoptosis in C4-2B cells (Fig 6B), Inhibition of MAPK pathway by U0126 (a MEKK inhibitor) showed no appreciable effect in preventing apoptosis in the same experimental conditions. c-JNK is known to phosphorylate and inhibit several proteins which play active roles in maintaining mitochondrial permeability barrier. By using the Mitotracker-red dye we observed that treatment with MK591 damages mitochondrial integrity and induces permeability-transition. The loss of mitochondrial membrane potential was evident by a significantly decreased red fluorescence in MK591 treated cells (Fig 6C).

### 3.7. MK591 triggers apoptosis in C4-2B prostate cancer cells involving down regulation of PKCε without inhibiting AKT

**Fig 7:**
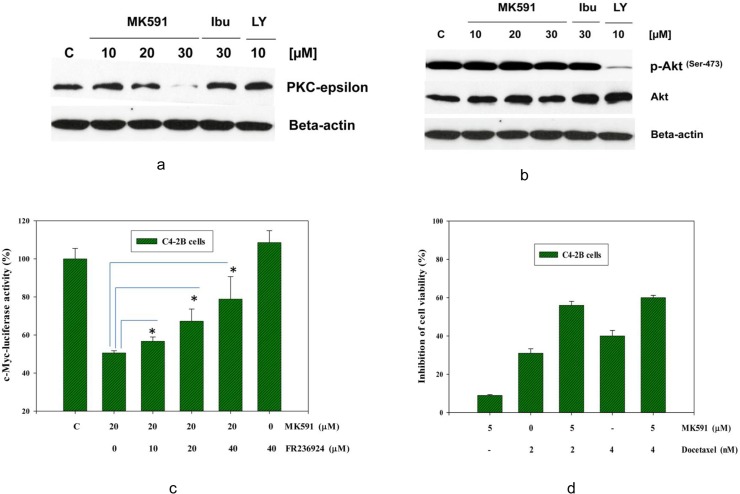
Effects of MK591 on Akt and PKC-epsilon. In (a and b), C4-2B cells were plated as in Fig 5 and then the cells were treated with MK591 as shown and incubated for 24 hours at 37^°^C. Cell lysate proteins were resolved by SDS-PAGE and levels of PKC-epsilon, or phospho-Akt (Ser-473) and total Akt were detected by Western blot using corresponding phospho-specific or regular antibodies. LY294002 (a PI3K inhibitor) was used as positive control which decreased phosphorylation of Akt but not the total protein of Akt. ***Note*:** MK591 does not affect the level of phospho-Akt or total Akt, but significantly decreases the protein level of PKC-epsilon. In (c), effect of FR236924 (an activator of PKC-epsilon) on MK591 treatment-induced loss of the transcriptional activity of c-Myc was detected by c-Myc-luciferase activity. ***Note*:** FR236924 significantly prevented the MK591 treatment-induced inhibition of c-Myc transcriptional activity. (d) Shows that MK591 and docetaxel synergistically affect the viability of C4-2B cells at low doses, Cells were plated as in Fig 1, and treated with MK591 and/or docetaxel as indicated for 48 hours. Cell viability was measured by MTS/PES Cell Titer assay as described in the “Methods” section.

The PI3K-Akt pathway plays an important role in the regulation of cell survival and apoptosis in many cell types [48–52]. However, recently we reported that 5-Lox inhibition-induced apoptosis in the androgen-sensitive LNCaP human prostate cancer cells occurs via inhibition of PKCε without inhibition of Akt [22,23]. We also found that a continuous activation of PKCε in prostate cancer cells occurs by signaling from OXER1, a G protein-coupled receptor (GPCR) for which the 5-Lox metabolite 5-oxoETE serves as the major ligand (30). Thus, we wanted to examine how MK591 affects Akt and/or PKCε while inducing apoptosis in the androgen resistant, bone metastatic C4-2B prostate cancer cells. Interestingly, we found that MK591 down-regulates protein levels of PKCε in C4-2B cells in a dose-dependent manner. However, MK591 does not affect the activating-phosphorylation of AKT (Ser-473) in the same experimental conditions (Fig 7A and 7B). Moreover, we found that FR236924, an activator of PKCε [53,54], prevents MK591 treatment-induced decrease in c-Myc transcriptional activity, suggesting that MK591 treatment-induced loss of c-Myc function in C4-2B cells occurs, at least partially, via down-regulation of the activity of PKCε (Fig 7C). Finally, we found that low doses of MK591 and docetaxel work synergistically to kill C4-2B cells (Fig 7D), which suggests that greater success can be achieved when these two compounds are used in combination at doses which are lower than the doses needed to be used individually as monotherapy.

## Discussion

Our current observations document that MK591, a second-generation leukotriene biosynthesis inhibitor, decreases viability of the bone-invading C4-2B prostate cancer cells (Fig 1). The C4-2B cell-line was derived from the parental LNCaP human prostate cancer cells, and represents advanced, castration-resistant disease. Based on the current state of understanding of prostate cancer as a disease, we believe that our findings are especially significant and of special interest in prostate cancer biology because these cells retain the characteristics of castration-resistant, bone-metastatic prostate cancer which is incurable by any therapeutic modalities currently available in the clinic. More interestingly, we found that MK591 does not affect normal, non-cancer human fore-skin fibroblasts (HFF) under the same experimental conditions. HFF cells, like many other normal body cells, do not express 5-Lox presumably because of promoter hyper-methylation [55–60]. Since MK591 does not affect normal, non-cancer cells, our findings also suggest that the anticancer effect of MK591 is largely based on cancer-specific molecular mechanism (s), and thus, should be devoid of adverse side effects. Further work is needed to test this concept by *in vivo* experiments.

Effect of MK591 on invasion and soft-agar colony formation by C4-2B cells at sub-lethal doses reminds that this agent may possess the capability of stopping the dispersal as well as new colony formation of prostate cancer cells at distant sites which are typical characteristics of the metastasis process (Fig 2). C4-2B cells are characterized to be castration-refractory and are equipped with special capabilities to invade bone [24,61]. Thus, our new findings of a remarkable effect of MK591 in preventing invasion and soft-agar colony formation of C4-2B cells brings hope for its use in treating deadly bone-metastatic prostate cancer which actually takes most of the human lives lost due to prostate cancer. The c-Myc oncogene is well-known to regulate various cellular signaling mechanisms that are responsible for enhanced growth, decreased apoptosis and resistance to therapy. Particularly noteworthy is the effect of c-Myc oncogenic signaling in promoting the metastatic ability of cancer cells via transcriptional up- or down-regulation of a range of other genes that participate in pro- or anti-metastatic cellular processes. Thus, we wanted to explore in greater detail the effect of MK591 in the regulation of c-Myc function. The dramatic inhibitory effect of MK591 on c-Myc function and expression of its target genes, support the concept that the anti-metastatic effects of MK591 may be mediated largely via down-regulation of c-Myc gene function (Fig 3). Recently, we found that 10058-F4, a chemical inhibitor of c-Myc, exerts anti-metastatic effects in LNCaP prostate cancer cells, suggesting a role of c-Myc in the metastatic progression of these prostate cancer cells (unpublished data). Further experiments are underway to explore this hypothesis.

How MK591-treatment triggers a dramatic loss of c-Myc protein in C4-2B cells is an intriguing question. The cycloheximide-chase experiment reveals that the MK591 treatment-induced decrease of c-Myc protein is not faster than a translational blockade by cycloheximide which suggests that a post-translational mechanism may not be responsible for MK591-induced loss of c-Myc protein (Fig 4). Loss of c-Myc protein by blockade of transcription by Actinomycin D and the actual decrease of c-Myc mRNA by MK591 confirmed the involvement of transcription inhibition as a mechanism in the regulation of c-Myc by MK591. Stat3 is one of the prominent members among the transcription factors to regulate c-Myc and is linked with 5-lipoxygenase activity in prostate cancer cells. Our observations of the inhibition of Stat3 transcriptional activity by MK591, and inhibition of c-Myc activity by Stattic (an inhibitor of Stat3) suggest that the down-regulation of c-Myc upon MK591 treatment occurs via blockade of Stat3-mediated transcription (Fig 4D,4E and 4F).

The morphological alteration in C4-2B cells (but not in HFF cells) reminded us that the predominant mechanism of cell death may be due to induction of apoptosis. We tested this hypothesis by analyzing cells for manifestation of apoptotic-features using standard apoptosis assays and observed that MK591 effectively induces externalization of phosphatidyl-serine, activation of caspase-3, cleavage of PARP, and degradation of chromatin-DNA to nucleosomal-fragments, which collectively document that MK591 kills C4-2B cells via induction of apoptosis (Fig 5). To understand whether the stress kinase, c-JNK/SAPK, plays any role in apoptosis we found a time-dependent increase in the activating phosphorylation of c-JNK in C4-2B cells upon MK591 treatment. Moreover, our observation of a significant inhibition of MK591-induced apoptosis by pre-treatment of cells with SP600125, an inhibitor of c-JNK activity, demonstrates that this type of apoptosis is mediated via induction of c-JNK activity (Fig 6). Previously we observed a rapid activation of c-JNK upon MK591 treatment in LNCaP cells which is prevented by exogenous addition of 5-Lox metabolite 5(S)-HETE in a dose-dependent manner, suggesting a critical role of 5-Lox in suppressing activation of c-JNK in prostate cancer cells [21]. A dramatic loss of mitochondrial membrane potential upon MK591 treatment indicates involvement of mitochondria in apoptosis induction which is also known to be promoted by activated c-JNK.

The Akt/PKB signaling is very well characterized to promote cell-survival and prevent cell-death via a range of defined anti-apoptotic mechanisms. However, our results show that MK591 triggers apoptosis in C4-2B cells without inhibition of Akt, suggesting that these cancer cells are equipped with additional (Akt-independent) survival mechanisms which are fundamental to their survival and may be regulated by metabolism of arachidonic acid via 5-Lox which is blocked by MK591 (Fig 7). An Akt-independent survival mechanism has been characterized in LNCaP cells using a variety of factors [62]. And recently, we explored a new survival/anti-apoptosis mechanism operating in LNCaP human prostate cancer cells which is promoted by arachidonate 5-Lox and is mediated via protein kinase C-epsilon (PKCε) which was found to be associated with a G protein-coupled oxo-eicosatetraenoid receptor (OXER1) for which 5-oxoETE (the active metabolite of 5-Lox) serves as the major ligand [30]. Our observation of the prevention of the loss of c-Myc function by the PKCε inhibitor, FR236924, documents that MK591 treatment-induced loss of c-Myc is mediated via inhibition of PKCε (Fig 7C). PKCε has already been shown to play a significant role in the development of androgen-independent prostate cancer and metastatic progression. Thus, our findings suggest that small molecule inhibitors, such as MK591 induces apoptosis in C4-2B prostate cancer cells which is independent of Akt inhibition, and that MK591 may prevent metastatic prostate cancer via down-regulation of PKC-epsilon and c-Myc oncogenic signaling. Since, low doses of MK591 and docetaxel work synergistically to kill C4-2B cells (Fig 7D), it appears that greater success can be achieved when MK591 is used in combination with docetaxel at doses lower than the doses currently used in the clinic as monotherapy.

It is interesting to note that under normal heath conditions, expression of 5-Lox is restricted to specific immune cells such as neutrophils, eosinophils, basophils and macrophages (not in T cells) where it plays a role in chemotaxis, whereas the vast majority of non-immune parenchyma body cells do not express 5-Lox unless inflammatory diseases occur, such as asthma, arthritis, psoriasis, and cancer [55–60,63–65]. Our recent findings revealed increased expression of 5-Lox in prostate tumors compared to adjacent non-tumor tissues (data not shown) which are consistent with data from previously published studies [66]. Altogether these findings suggest that 5-Lox may play an important role in prostate cancer development and progression. Thus, the 5-Lox pathway is emerging as a promising target for therapeutic development against prostate cancer. Putting all together, our findings indicate that MK591 possesses all major attributes of a standard anti-metastatic agent with significant cancer-selective effect, and suggest that MK591 may turn out to be an effective agent for therapy of castration-resistant, bone-metastatic prostate cancer. Though details of the molecular underpinnings of the anti-metastatic action of MK591 are unknown at this time, it gives us an opportunity for further exploration to better understand the signaling mechanisms involved by *in vitro* and *in vivo* experiments.
